# Hope is everything – an hermeneutic phenomenological study on the lived experiences of individuals with adrenocortical carcinoma

**DOI:** 10.3389/fendo.2026.1829424

**Published:** 2026-07-08

**Authors:** Phillip Yeoh, Jackie Sturt, Wladyslawa Czuber-Dochan

**Affiliations:** 1 Florence Nightingale Faculty of Nursing, Midwifery & Palliative Care, King’s College London, London, United Kingdom; 2 Department of Endocrinology & Diabetes, The London Clinic, London, United Kingdom

**Keywords:** adrenocortical carcinoma, hermeneutic phenomenology, hope, lived experiences, rare cancer, resilience, survivorship

## Abstract

**Background and objective:**

Adrenocortical carcinoma (ACC) is a rare and aggressive malignancy of the adrenal glands, characterised by high recurrence rates and poor survival outcomes. Living with ACC is associated with significant uncertainty and psychological burden. This study aimed to explore the experiences, impacts, and perceptions of people living with ACC to inform the development of comprehensive, person-centred care.

**Methods:**

A qualitative study underpinned by Van Manen’s hermeneutic phenomenological methodology was undertaken. Semi-structured interviews were conducted, informed by Mullan’s concept of survivorship, Bowen’s Family System Theory, and the concept of resilienc*e*. These frameworks guided both data collection and analysis.

**Findings:**

Twenty-one participants (12 women, 9 men; mean age 50, range 28–74) from the USA, Canada, UK, Europe, Nigeria, and Australia took part in interviews totalling 19 hours and 34 minutes. Five themes and 11 subthemes emerged. Living with ACC was described as an existential challenge, as participants navigated the reality of limited survival alongside demanding, life-prolonging treatments. The pursuit of survival was likened to a rollercoaster, requiring sustained support and continuous adaptation. For some, these challenges became overwhelming and led to thoughts of suicide. Participants highlighted a strong need for hope, understanding, and compassionate, ongoing care to help them cope, adapt, and rebuild their lives.

**Conclusion:**

ACC is a complex and life-altering condition that requires careful balancing of treatment burden with quality-of-life considerations. Current healthcare systems do not adequately meet the needs of this population. Future research should focus on developing and evaluating interventions to better support individuals living with ACC and mitigate the psychological and practical impacts of the disease.

## 1 Background

Adrenocortical carcinoma (ACC) is a rare and aggressive malignancy originated from the cortex of the adrenal glands. Rare cancers, defined as those with an incidence of fewer than 6 cases per 100,000 people annually, account for approximately 24% of all cancers in the European Union (EU) (1). The global incidence of ACC is estimated to be 0.7–2 cases per million people per year in adult populations (1, 2). According to the European Network for the Study of Adrenal Tumours (ENSAT) staging system, the 5-year survival rate for individuals with stage IV disease can be as low as 5.9% (3). Furthermore, patients with a tumour proliferation index (Ki-67) of ≥20% have a median recurrence-free survival of only 9.4 months (4).

Individuals with ACC frequently present with symptoms related to excess adrenocortical hormone secretion, including hypokalaemia, hypertension, male pattern baldness, hirsutism, virilization, menstrual irregularities in women, and gynaecomastia in men (5, 6). Approximately 13% of patients are initially misdiagnosed as having other malignancies, contributing to delays in appropriate treatment (7). Surgical resection remains the cornerstone of management, with open adrenalectomy demonstrating superiority over laparoscopic approaches in achieving complete oncological resection and reducing recurrence rates (8).

Adjuvant mitotane therapy is recommended for patients at moderate to high risk of recurrence and is typically administered for a minimum of two years and up to five years (5, 9). Therapeutic efficacy requires maintaining plasma mitotane levels between 14-20mg/L, which can be challenging due to dose-related toxicity and individual variability in metabolism (10). Adverse effects commonly include endocrine, gastrointestinal, hepatic, haematological and neurological complications (11). For patients with advanced or progressive disease, combination chemotherapy with etoposide, doxorubicin, and cisplatin (EDP) is recommended as first-line treatment (5, 12).

Despite established clinical guidelines, there remains a significant gap in understanding the lived experiences of individuals with ACC (5). Evidence suggests that patients experience substantial physical, psychological, social, and functional burden, with long-term survivors continuing to report fatigue and emotional distress (13–15). Given the limited qualitative research in this area, this study aims to explore how individuals with ACC experience and perceive their condition, and how it affects their everyday lives.

## 2 Research design and methods

The study adopted a qualitative design grounded in hermeneutic phenomenology, an approach concerned with exploring how individuals experience and make sense of specific experiences (16). Hermeneutics focuses on the interpretation of meaning as expressed through experiences, narratives, actions, and social contexts. Van Manen’s hermeneutic phenomenological approach extends to interpreting its deeper, subjective significance, emphasising reflection, openness, and engagement with participants’ accounts (17, 18).

The researcher adopted a reflexive stance throughout the study, remaining attentive to participants’ perspectives, data meanings, and the researcher-participant relationship during data collection and analysis. The researcher also previously conducted a systematic review on the lived experience of ACC (15). This, combined with his 25 years of clinical experience working as a Consultant Nurse in Endocrinology caring for people with ACC, informed and shaped the reflexive and interpretative process. Mullan’s concept of cancer survivorship, encompassing the acute, extended and permanent phases of survivorship, was used as a sensitising framework to explore participants’ experiences across different stages of living with cancer (19). In addition, Family Systems Theory, which examines how family members influence and respond to one another’s experiences, informed the exploration of relational and social dynamics (20, 21). The concept of resilience was also drawn upon to enhance the depth and richness of both data collection and analysis, enabling a more nuanced understanding of how individuals with adrenocortical carcinoma adapt to, cope with, and make meaning of their illness over time. Ethical approval for the study was obtained from King’ College London (Reference Number: HR/DP-21/22-29110). All participants provided written informed consent prior to participation, and the study was conducted in accordance with the principles outlined in the Declaration of Helsinki.

## 3 Sample and recruitment

Participants were recruited through advertisements placed on the websites of international ACC patient support groups (ACC Support UK and Adrenal Cancer Warriors Group), as well as through The London Clinic (UK). Participants were informed that the researcher had more than 25 years of clinical experience in endocrine cancer nursing, including a particular professional interest in the care of individuals living with ACC. This background was disclosed to ensure transparency regarding the researcher’s prior knowledge, assumptions, and commitments, and to enable participants to consider how these might influence the research relationship and interpretive process. The study was presented as part of an ongoing commitment to deepening understanding of patients’ lived experiences and to applying these insights to enhance clinical practice, supportive care, and future research for those affected by this rare cancer. Participants were also advised that the study was being conducted as part of the first author’s doctoral research. They were therefore aware of the researcher’s professional background and motivation for undertaking the study, as well as the potential influence of this positionality on data generation and analysis.

Purposive sampling was employed to recruit individuals with a confirmed ACC diagnosis who were aged 18 years or older and able to communicate in English. Individuals diagnosed with other types of adrenal tumours were excluded from the study. To enhance the richness and diversity of participants experiences, a sampling framework was developed based on key characteristics including sex, age, duration of living with ACC, marital status, hormonal status, ENSAT stage, Ki67 index, past and current treatments, disease response, and comorbidities. These data were collected through a demographic form completed during the screening phase. Potential participants were contacted by the researcher to assess eligibility and to ensure inclusion of individuals who met the principle of maximum variation. Sample adequacy was determined using Malterud et al.’s concept of information power (22), which considers the study aim, sample specificity, quality of dialogue, analytic strategy, and the richness and relevance of the data obtained.

## 4 Data collection

Data were collected through one-off, one to one, semi-structured, individual in-depth interviews conducted by the researcher. In two instances, the supervisor (WCD) assisted with the interviews. Interviews were conducted in participants’ homes between July 2022 and August 2023 in accordance with participants’ preferences, either via Microsoft Teams (*n* = 20) or by telephone (*n* = 1). An interview topic guide (**Supplementary File 1**) was developed, piloted, and refined with input from patient and public involvement (PPI) contributors to ensure relevance, clarity, and sensitivity. Fieldnotes were taken during and after the interviews to support the data analysis.

## 5 Data analysis

All interviews were transcribed verbatim and coded by the researcher. The transcripts were imported into NVivo (version 20) to facilitate data management and organisation. Data analysis was conducted over 24 months and informed by Braun and Clarke’s reflective thematic analysis (23) alongside van Manen’s hermeneutic phenomenological approach (17, 18). The integration of these complementary analytic frameworks provided a structured yet flexible process for data familiarisation, coding, interpretation and theme development. This approach enabled the identification and interpretation and exploration of the phenomenological meanings embedded within participants’ accounts, which were iteratively developed into themes and subthemes to provide a rich understanding of their lived experiences.

## 6 Findings

The interviews ranged in length from 25 to 87 minutes, with a mean duration of 55 minutes, resulting in a total of 19 hours and 34 minutes of interview data. The analysis process generated 296 initial codes, which were iteratively reviewed, refined, and organised into overarching themes.

In total, 28 individuals with ACC expressed interest in participating, of whom 21 were included in the study. Seven participants were excluded due to poor health (n=2), loss of contact (n=4), or having similar disease trajectories and treatment experiences to those already recruited (n=1). All participants had a confirmed diagnosis of ACC; three male participants also had additional cancer diagnosis.

A summary of participants’ demographics and clinical characteristics are presented in **Table 1**, with more detailed information provided in **Supplementary Table 2**. The sample comprised 19 Caucasians, one North African, and one Black African participant. The mean age at diagnosis was 44 years (range 22–67 years), and the mean duration of living with ACC was six years (range 10 months to 21 years). Sixteen participants self-reported hormone excess and were in disease remission at the time of interview.

**Table 1:** Participants demographic and clinical characteristics.

Characteristics	Category	Participants (N = 21)
Sex	Male	9
	Female	12
Marital status	Married/partnered	16
	Single/widowed	5
Age of ACC onset in years	20–30 years	4
	31–40 years	4
	40–50 years	6
	51–70 years	7
Age at recruitment	20–30 years	1
	30–40 years	6
	40–50 years	4
	51–75 years	10
Duration since diagnosis	Up to 2 years	5
	2–5 years	6
	5–10 years	4
	10–21 years	6
ENSAT tumour stage	Not known	9
	I	0
	II	5
	III	2
	IV	5
Ki67%	Not known	7
	<10%	2
	10-19%	4
	>20%	8
Occupation	Unemployed	6
	Employed	8
	Retired	7
Previous/current treatment*	Surgery	20
	Mitotane	18
	Chemo/immunotherapy	4
	Ablation/radiotherapy	4
Disease status of those taking mitotane at the time of interview	Remission	5
	Active	4
ACC disease status	Remission	16
	Active	5
Self-reported comorbidities*	Adrenal insufficiency	18
	Hypothyroidism	7
	Clotting disorders	4
	Chronic pain	4
	Hypercholesterolaemia	3
	Obesity	2
	Erectile dysfunction/vaginal tightness	2
	Hypertension	2
	Diabetes	1
	Osteoporosis	1

*It should be noted that not all participants provided complete information regarding their treatments or comorbid conditions.

## 7 Themes and subthemes

The findings are presented as five themes and eleven subthemes (**Table 2**), reflecting the diverse meanings and impacts of living with ACC. The themes are illustrated using verbatim interview extracts, accompanied by participants’ pseudonyms and ages (**Tables 3**–**5**).

**Table 2:** Study themes and subthemes.

Themes	Subthemes
1. Existential conundrum of living with ACC	1.1 Living in uncertainty 1.2 Life on a knife edge 1.3 Coping and protecting wellbeing
2. Inadequate care and support	2.1 Loneliness and being left behind 2.2 Tensions within self and with others
3. Rollercoaster life	3.1 Enduring the disease burden 3.2 Wading through the trauma
4. Anchoring ways for survival	4.1 In the illusion of control 4.2 Knowing me, knowing ACC
5. Living and moving forward	5.1 Finding perspectives and priorities 5.2 Create moments of happiness

**Table 3:** Theme and subthemes 1.

Themes/subthemes	Supporting data (participant pseudonym & age)
Theme 1 Existential conundrum with ACC	“The surgeon said basically you have a really rather large adrenal tumour, and it’s pushed your right kidney down and the liver so it’s really quite big. It could be in the liver. It might not, he can’t tell from the scan. But he also just said that the tumour had grown up the IVC [inferior vena cava] so that was a problem.” (Jack, 28) “I got all my affairs in order beforehand. Made sure all my t’s were crossed and I’s dotted so my wife could continue in life after me.” (David, 62) “I did so many things with my life. I have no regrets. Whatever it was, I got it. But I didn’t want to suffer. And in this point, I want to know what’s the options are. And in other words, when I went out of hospital, then informed myself about this on how, which are they ways to die. In my country, there is an option of self-assisted suicide. So, I became a lifelong member of EXIT, which is the largest self-assisted suicide group. “ (Kevin, 50) “As it’s my body, I made my own decision; I told them that I was fine to take medication all my life, and I prefer that. We say, in fact, “Damocles’ sword”. “ (Sophie, 54, F) “There’s sort of the feeling of, is all this effort really worth it. If all we had just spent on being alive instead of on living, you know I wouldn’t say that most days feel that way. There’s definitely several days where I’m like, why am I doing this? Like this can’t be, is this worth doing this all day every day? Like we’re prolonging my life as long as possible so that this is what I can do every day?” (Kate, 41) “I’ve got it for life until it kills me. Basically, I don’t think I’ll ever be cured. It’s just a question of living with it and dealing with it and waiting until it beats me.” (Lucy, 63)
Subtheme 1.1 Living in uncertainty	“I started getting more symptoms like polycystic ovarian syndrome, like extra body hair. I never had acne before I got acne. And by the time I started losing my scalp hair and to a great extent, the ultrasound found a six-centimetre lump, and nobody could tell what that was.” (Lucy, 63) “I did go a bit crazy and very hyperactive, as if I’d got massive amounts of energy. I couldn’t sit still. I needed to do things, and my mood swings were really high or low. I’d get quite emotional with my bad mood swings (….). I never heard of Cushing’s. Cushing’s to me was something that horses or dogs got not human beings. And I didn’t know what it was’ “ (Carrie, 52, F) “At the start, I remember I was just trying to go online and that was like a rabbit hole. It just led to a lot of depressing information about survival rates. I think pretty early into my treatment I just stopped looking at that.” (Lisa, 32) “The fourth chemo round was really bad. It was a full day of poison in me. I went septic and was rushed into the hospital. They told my wife to get my family around me because they didn’t think I was going to make the night. But yeah, the chemotherapy was horrible, horrible, horrible. Really disgusted.” (Wally, 63) “It was exhausting. It’s very difficult to separate now what was post ops recovery. But I was on stonking doses of hydrocortisone, like 50 or 60 milligrams a day as normal maintenance. And then I would get sick, (…) and going into hospital regular. I was probably admitted with sepsis and adrenal crisis every three months. Both, you know a good long period that probably didn’t settle down until about five years ago.” (Tom, 39) “Over the last 10 years there’s been lots of different doctors, lots of different opinions, and lots of different proposed kind of prognosis, and how long I’m going to live. Some of them said you’ll be lucky to make three years some five and some just see how it goes. So, we just went with the “see how it goes” thing which is what I’m doing now to see what you can do to keep going.’’ (Rob, 43)
Subtheme 1.2 Life on a knife edge	“I was very depressed that this oncologist that I went to see dismissed me (…). In fact, he wasn’t the only one because we phoned up a few countries cancer centres and spoke to them on what they would do. None of them had any solutions. It was all doom and gloom from everybody,” (Betty, 74) “But I remember that in the intensive care after surgery, the doctor told my mom that I had almost died. And it was a close one. That’s not something that a mom should hear.” (Emma, 38) “I’m no longer a human being; I’m a time bomb.” (June, 39) “I am going to die, I reflected, I think that’s the worst about it.” (Kevin, 50) “It got to the point where I think I was planning to end my own life. You know, there are different stages of suicide. I think this was like the like the highest one where you’ve made a plan and stuff.” (Jack, 28) “I think like with any cancer, I will never not think about it, that it may come back.” (Jim, 67)
Subtheme 1.3 Coping and protecting wellbeing	“There’s a lot of suffering. So yes, I try to avoid it cause there’s nothing I can do about it. I don’t want to feel too emotional about it. Emotional is not going to help. It can only make things worse.” (Sue, 61) “I try to block it out on a permanent basis. I don’t think about it anymore. It’s always there but it’s not going to ruin my life now.” (Wally 63) “I always say I’m the luckiest unluckiest person. I’m lucky because I had a very good GP who said something’s not right. Instead of taking me for an ultrasound, sent me for a CT chest abdomen and pelvis, which is probably not the right thing to do for a fibroid. And it [ACC] was caught by an accident.” (Muriel, 50). “Well, my endocrinologist, he saved my life. First on mitotane. The two of them went together.” (Betty, 74) “I think that everyone should be followed by a therapist or something of the kind, psychologist, because it changed your life. I’m very angry as well because life choices were forced to me. And it’s very in you have to deal with a lot of stuff. So, I think that that is something should be done.” (Emma, 38) “I find that sort of comforting. Because sometimes I think, oh well how big were their tumour again? You know that girl’s three years post-surgery and her Ki67 was as high as mine. So that’s okay. I’m going to be okay.” (Muriel, 50)

**Table 4:** Theme and subthemes 2.

Themes/subthemes	Supporting data (participant pseudonym & age)
Theme 2 Inadequate care and supports	“I think for the average person, the GP would not have found this cancer, just on manual examination. They would’ve been dead before they could get an NHS appointment. “ (Betty, 74) I think a lot of people with cancer have to come to terms with life and death. You know, it changes your life. And I think mine has allowed me to, look at how can I help others with this? And possibly because I didn’t have help that I think it’s given me some direction that I would not have had otherwise.” (Jim, 67) “Because you don’t know, there’s no one, and it’s not the doctors’ fault because they don’t know what the right thing to do is there’s not enough evidence about it. I think the thing that would make the whole process easier would be if there was significantly more research and like, you know, proper trials as to how effective mitotane is. And that’s just doesn’t really seem to exist out there.” (Mary, 35) “Interviewer: Did you have anyone to talk to when you felt afraid at that time? Jim: I did not. Interviewer: Was there anyone in the family you talked to? Jim; Not really, no. It was very lonely.” (Jim, 67)
Subtheme 2.1 Loneliness and being left behind	“I had meetings with so many specialists, and everyone would say, you’re so rare. We’ve never heard of this before. And you know, it doesn’t help when people say that over and over again to you. “ (Muriel, 50) “But nobody can give you a prognosis with all this, but they could only tell me what they knew scientifically. Couldn’t do anything else (…). It [mitotane] cost in the thousands, and I can’t remember, it was a horrific amount every month. I don’t know what other people do. I did ask the GP, they weren’t all interested or the health service, not interested” (Betty, 74) “It’s understandable because drug companies are not going to spend a huge amount of money for a few patients. And that’s a fact of life.” (Stan, 69) “I feel frustrated that that I don’t have a choice. We don’t have any other solution, and people are not looking for other solution. We discovered ACC on dogs, and that we didn’t change anything for 40 years.” (Emma, 38) “One of the MacMillan nurses come around, and she could see I was at home on my own sort of going over what had happened. And she said there is a day hospice (.) She said on a Tuesday, they’re all your age (.) you can speak to them better than you can speak to your wife or your friend (.) someone who’s had the same experience (.) But the only thing was as I had met these people, they went on and died. And after about the six months, I was one of the only people who was originally there, you know, and I thought this is getting depressing.” (Wally, 63) “I just couldn’t get a job as a permanent emergency doctor because I had too much time out and the system really didn’t like doctors who were part time.” (Tom, 39)
Subtheme 2.2 Tensions with self and with others	“I think sometimes you can get frustrated that this has happened to you. I kind of look back and think what did I do wrong? Where did I go wrong? What did I do? Was it something that I ate? Was it something that I did wrong? Is someone punishing me you go you answered that. All those questions in your mind” (Carrie, 52) “I couldn’t do nothing. My whole life changed. I couldn’t go out. I just sit there, and the children were trying to do their exams. And it was playing on their mind that their dad was at home not knowing what his future was. And that made me sad as well. But yeah, it was a tough time. Very tough.” (Wally, 63) “My oncologist said to me do you feel that you and your husband would benefit from some counselling? And I said no, because I’m quite hardcore lady (…), quiet toughie and not really good at talking to counsellors about my problems. But my husband said no, I think it’d be a good idea. And I went along more for him to this meeting. And it was quite funny, because I was kind of okay, but my husband started crying and then I’m shaking, and it’s kind of woke me up to realise what he’s going through. Not me if that makes sense. And then I thought I had to be strong for my husband. And it was really weird. And he because he started crying. I then started crying. And it was a bit of a moment.” (Carrie, 52) “I’ve got two distinct camps, one in denial and one that just wants to ignore, which, you know, leaves a lot of people who need support. (…) It is an odd place to be in, where you feel like you have to be the emotional support to people who this is not happening to.” (Kate, 41) “You have to be really real. I think that because doctor doesn’t know because sometimes you are the first case. They just read the scientific to you and they don’t tell you, or maybe they don’t want to you to be afraid. I’m one of the people that wants to know everything. That’s why I read everything.” (Emma, 38).

**Table 5:** Theme and subthemes 3, 4, and 5.

Themes/subthemes	Supporting data (participant pseudonym & age)
Theme 3 Rollercoaster life	“Well, it wasn’t actually the cancer which affected me physically. It was the medication. I took the mitotane, which affected things.” (Betty, 74) “The mitotane therapy was the hardest part. Like I feel surgery in that regard, I could just soldier through it. But mitotane therapy was just an ordeal.” (Lisa, 32). “Emotionally, it’s a bit of a roller coaster because I get scanned every three months. You get scanned, you don’t look forward to the scan because you know it’s coming, then you have to wait for the results. And then that can take three weeks sometimes for someone to ring you to tell you what’s going on. As soon as you get the results let’s just say I’m clear. I get the results. And then it’s almost time for the next one.” (Lucy, 63) “The worst thing is for my children really that they’re starting to think that I might not be there when they’re old. And my wife as well she’s just very upset about the whole thing all the time. But for me personally, I’m alright. I just worry about what other people think and feel about it.” (Rob, 43) “If I hadn’t had the disease, I would be a consultant in emergency medicine, but I probably wouldn’t be living in the city. A lot of other things about my life would be different. And you get very frustrated at that because it made a major change to my life and what I was doing. But I have now handled something that was allegedly unsurvivable when it was diagnosed.” (Tom, 39)
Subtheme 3.1 Enduring the disease burden	“If you look at mitotane effect, it’s normal, it’s amazing, it’s just toxic medicine. But it keeps you alive. I’m going to take it; I can do it.” (Kevin, 50) “And yet this poison you’re taking, it’s very weird psychologically to be actively choosing to take something that makes you feel really, really ill.” (Mary, 35) “When I had gall stones, I was constantly in hospital and the whole process is just incredibly painful. I can be there all day, and they don’t really understand it and it’s really frustrating because I’m completely spaced out because I have gone into adrenal crisis. And I’ve discharged myself before in hospitals because they just didn’t know what they were doing at my local hospital.” (Carrie, 52) “You have invisible illnesses, and I have three, ACC, adrenal insufficiency, and chronic pain. And you have to deal with that (…). In the pain, it’s a real pain and it’s a real pain for the soul, physically in the body and in the soul.” (Emma, 38) But no nausea. Oh, I always have nausea, so I just, sometimes I forgot that I have nausea. (Muriel, 50)
Subtheme 3.2 Wading through the trauma	“For the first week when I came out intensive care, it was quite emotional because all of those sorts of dreams were floating around in my head still (…) I struggled with them (…). I think it’s quite common to have this sort of strange experiences. And basically, I wrote them all down on a piece of paper and what I could remember all the recurring ones. And I put them in a box, and I was out walking the dog, and I took them with me, and I dug a hole in the forest, and I buried them. And that was the end of it.” (Stan, 69) “The negative aspect is always going to be there, you know, the sea glass of exercise trauma. The physical and psychological side effects took a long time to deal with.” (Tom, 39) “I don’t talk because I don’t want the burden with my problems.” (Wally, 63) “And you don’t really want to dwell on it. You want to like move on with life. And I think it’s probably made me like, more resilient and stronger as a result.” (Mary, 35)
Theme 4 Anchoring ways for survival	“It’s really a case of it is what it is, get on with it! That’s the only way I can describe it really. I know it sounds a bit flippant, but you can’t spend your life worrying about what might happen. It is what it is. Just get on with it and do as much as you can while you can.” (Stan, 69) “You take several tablets; you just get on with it. It’s part of your routine and that’s it. Like you have your coffee.” (Betty, 74) “I approach it with a positive attitude that I’m being well monitored looked after and well cared for. Hence why I’m still alive now because you know, 10 years plus is quite a good prognosis really for adrenal cancer.” (Lucy, 63) “I’m an enigma, especially because I had a second, according to an ACC expert and everybody else think that the second experience with ACC was actually a new experience different from the first experience because of the seven-year gap. With ACC, it was more internal spiritual, and I was alone with my spirituality.” (Jim, 67) “I just count my lucky stars in that respect. And my dad’s saying a prayer for me every day.” (Wally, 63)
Subtheme 4.1 In the illusion of control	“Everyone and me in particular, needed that illusion of control over what we were doing in our life” (Tom, 39) “I didn’t really do much in that three and a half years other than just try and cope with all of the side effects of the mitotane. In the end, when you get experience with it, you learn how to play the mitotane in the way of what you eat and what you do and so you learn how to manage it better.” (Carrie, 52) “I don’t want to get me down. Then you’d be trampled on you, you’d be dead. Or anything can happen. It’s simple. I just believe in people being in charge of the situation. I like to be in charge of situations.” (Betty, 74)
Subtheme 4.2 Knowing me, knowing ACC	“When I first found out about the disease and you know, the high mortality rate quite quickly after diagnosis and surgery. Obviously, that was quite concerning, but I’m now sort of 10, 11 years post-surgery, (…), I think it weighs less on my mind now than it did when I was 60.” (Stan, 69) “I listen to my medical experts tell me and I follow their instructions. However, I am not slavish in following it [mitotane treatment].” (James, 69) “As it’s my body I made my own decision, I told them that I was fine to take medication all my life, and I prefer that. We say in fact Damocles sword.” (Sophie, 54) “In terms of survival, it comes down to the original surgery and the original post-op period and once you get past that, your prognosis improves dramatically.” (Tom, 39) “My advice would be to do what you think is right and don’t get swept up with what everybody else is doing. Do what you think is right for yourself and do what you would do normally and don’t spend hours on the internet and hours on Facebook.” (Stan, 69)
Theme 5 Living and moving forward	“I don’t want to explain to people because I don’t want to be pitied or to appear like a victim. But it’s just the way it is, people don’t understand that.” (Emma, 38) “I’ve just treated life as goals, like seeing my daughter graduate, becoming a grandma, hitting my 60th birthday, seeing my son get promoted get married.” (Lucy, 63) “It will always be a part of my identity, but I don’t want to make it fully my identity as a cancer survivor and it helped me to not focus on that.” (Lisa, 32) “But now, I feel like I’m out of it. Now. I knew what will happen. If symptoms come back. It’s going to be meeting me again. I think we’ve developed a way of handle that as best as possible. And if that comes back then, you know so be it. In the meantime, it’s about time I got off my backside and started living again’.” (James, 69)
Subtheme 5.1 Finding perspectives and priorities	“I’m still here. Mortality statistics for ACC were awful. The fact I’ve survived at all was a miracle.” (Tom, 39) “The one most important thing that you live for the day. You don’t think too much of the future. I’m not going to think too much. Just live for the day. Every day is like another day.” (Sue, 61) “**As a patient, hope is everything and a priority. If they could have given me hope all the time, that would’ve been a lot better**.” (Wally, 63) “Well, it’s [hope] given me direction to help others, so I think it’s, it’s made me look at what is important in my life.” (Jim, 67) “At some stage I know that I probably have to make a decision about whether I would want chemo or not. Ought that be the decision that I would take. It’s not a given that I would say yes, I would. Because I may make the decision if things carry on and say I get to sort of my mid-seventies without having to do it, I might make the decision that actually no, I really don’t want to do this when I’m 70 +.” (Stan, 69)
Subtheme 5.2 Create moments of happiness	“Living through it [life] more intensely than like an average person would.” (Lisa, 32) “I always create moment of happiness because I know life can end for everybody. I have more hope than before. But I know, in the end, I can die. Therefore, I’m really enjoying life as much as I can. And I am grateful for life in circumstance I have got.” (Kevin, 50) “There’s nothing obvious about my cancer experience. It doesn’t stop me from golfing or from traveling or things that I like to do.” (Jim, 67)

### 7.1 Theme 1: existential conundrum of living with ACC

The diagnosis of ACC profoundly altered participants’ sense of existence, evoking complex emotional responses including shock, depression, confusion, denial, feelings of being lost, disappointment, difficulty processing their circumstances, and a pervasive sense of hopelessness. For many (e.g., Jack and David), the experience of living with ACC triggered a heightened awareness of mortality and prompted reflection on the meaning life and the impact of their illness of those around them (24).

Several participants, including Sophie, likened their experiences of ACC management to living under the ‘Sword of Damocles’ - struggling to survive while constantly facing the threat of disease progression and treatment-related risks. In Roman mythology, the Sword of Damocles symbolises the precariousness of fortune, suspended by a single horsehair above Dionysus’ throne. This metaphor aptly captures the ongoing uncertainty faced by individuals living with cancer: although granted continued life, they remain burdened by fear of recurrence, malignancy, and the long-term consequences of treatment (25, 26).

For some participants such as Kevin and Kate, these existential challenges were overwhelming. Two male participants (Kevin, Jack in subtheme 1.2) described experiencing suicidal thoughts in the early stages following diagnosis. Among those within two years of diagnosis, three participants reported that the disease burden was so profound that it led them to question the purpose and meaning of their lives. These narratives highlight the depth of existential distress and uncertainty associated with ACC (**Table 3**). For individuals such as Lucy, who had lived with active disease for over a decade, the persistent risk of recurrence remained ever-present, reinforcing ongoing fears that they might never be fully cured.

#### 7.1.1 Living in uncertainty

This theme captures participants’ experiences both prior to diagnosis and throughout their survivorship with ACC. Symptoms associated with hormone excess, particularly Cushing’s syndrome, intensified feelings of uncertainty and fear, contributing to heightened anxiety in Lucy and Carrie. The rarity of ACC further compounded this uncertainty, as healthcare professionals (HCPs) often experienced difficulties in recognising and managing the disease, which was frequently masked by features of hormone excess or misattributed to other conditions.

Limited availability of reliable information about ACC (e.g., Lisa’s quote), coupled with exposure to survival statistics found online, further heightened participants’ anxiety. ACC was experienced as both an affective and cognitive challenge: emotional responses were shaped by how participants interpreted their experiences, while their cognitive processing of the illness influenced their emotional wellbeing. For many, Cushing’s syndrome was particularly difficult to comprehend and had a profound emotional impact as described by Carrie.

Adjuvant treatments, including mitotane and chemotherapy, were described as intensive and burdensome, often associated with severe and potentially life-threatening side effects, as reflected by Wally and Tom. The development of adrenal insufficiency (AI) added further complexity, significantly affecting daily life and long-term wellbeing. Uncertainty regarding the possibility of cure and ambiguity about the future remained persistent concerns for participants. This enduring sense of uncertainty has also been reported among individuals living with other rare cancers (13, 27). For participants such as Rob and Lucy’s description in theme 1, whose treatments were unsuccessful or who experienced ongoing disease progression described a loss of trust in available treatment options and were forced to confront the realities of living with a chronic and life-limiting condition.

#### 7.1.2 Life on a knife edge

Participants such as Betty described confronting profound existential threats, particularly in the early stages following diagnosis and throughout their lives with ACC. Betty, Emma, June and Kevin spoke of having to come to terms with the risks they faced and preparing themselves for what lay ahead, including the possibility of death. For those who overcame the initial shock, the realities and consequences of treatment became increasingly apparent. Some participants described reaching a point where they sought to regain a sense of control by deciding for themselves whether they wished to continue living.

For example, the impact of mitotane treatment on renal function during the early phases of his survivorship led Jack, who feared permanent dialysis due to having only one kidney, to contemplate suicide. For these individuals, thoughts of death were ever-present, and living with ACC was experienced as akin to carrying a death sentence (e.g., Jim’s quote). The burden of the disease extended beyond the individuals themselves, profoundly affecting their loved ones. Participants described the emotional, psychological, and physical toll their illness imposed on family members, further intensifying the weight of their experience.

#### 7.1.3 Coping and protecting wellbeing

The participants such as Sue and Wally described employing a range of coping strategies to manage their illness and protect their wellbeing. Coping was understood as a conscious effort to reduce distress and manage the emotional, psychological, and social impacts of ACC. Strategies included stoicism, denial, emotional withdrawal, acceptance of the illness, and the cultivation of a positive mindset.

Individuals who were older, had lived with ACC for longer, or had experienced previous or concurrent cancers seemed to cope better, drawing on prior experiences of illness and adversity. In contrast, those who were younger or newly diagnosed often described greater difficulty adjusting to the diagnosis and its consequences. Many participants such as Muriel and Betty attributed their survival partly to luck but also emphasised the critical role of specialist care and the expertise of their ACC clinical teams. The importance of psychological support was strongly highlighted by Emma, with several participants noting a lack of access to counselling or formal emotional support. Peer support, particularly through ACC support groups, was described as invaluable as described by Muriel. These communities provided a sense of connection, understanding, and shared purpose, enabling participants to find hope, gain knowledge, and support others facing similar challenges.

### 7.2 Theme 2: inadequate care and support

Human beings are inherently relational, with the capacity to form meaningful connections that foster understanding, purpose, and a sense of belonging (18). For participants in this study, however, these relational experiences were often compromised. Betty, Jim and Mary described their interactions with the world around them, including healthcare systems, professionals, and wider society, were frequently characterised by a lack of awareness, knowledge, and understanding of ACC. This was compounded by limited research and insufficient systemic structures to adequately support their complex needs (**Table 4**). Participants described how these gaps in knowledge and care negatively affected their experiences, contributing to feelings of isolation, frustration, and abandonment. The perceived inadequacy of support was often interpreted as a lack of compassion and, at times, a diminution in the quality of their clinical care. As a result, participants felt that their needs were not fully recognised or addressed, further intensifying the emotional and psychological burden of living with a rare and life-limiting condition.

#### 7.2.1 Loneliness and being left behind

For many participants, the first point of contact for help and support was the healthcare professional who diagnosed their ACC, typically their general practitioner, local endocrinologist, or oncologist. However, approximately half of these centres had limited experience in managing ACC, as experienced by Muriel and Betty. As a result, participants frequently turned to online sources in search for information, where they were confronted with bleak survival statistics and a lack of reliable guidance. This limited awareness and expertise in ACC care was perceived as detrimental to both their health outcomes and overall wellbeing.

Eleven participants who survived five years or longer received care from specialist centres with expertise in ACC, highlighting the positive impacts of specialist knowledge and coordinated care. In contrast, the high cost of treatments, restricted treatment options, and limited access to return-to-work support contributed to feelings of frustration, isolation, and helplessness, which was experienced by Betty, Stan, Emma and Tom.

Living with ACC often required individuals to endure significant physical and emotional suffering, which many felt was inadequately acknowledged or addressed within healthcare systems. For those who sought peer support through cancer support groups, the experience could be emotionally complex, as witnessing the illness or death of other members compounded their own distress, such as described by Wally. Only 38% of the participants were in employment at the time of the study, reflecting the substantial impact of ACC on functional capacity and quality of life. Collectively, these experiences contributed to a pervasive sense of loneliness and of being left behind or marginalised, both socially and within healthcare systems.

#### 7.2.2 Tensions with self and with others

Living with ACC intensified the participants’ awareness of their human condition and reshaped their relationship with others. The human condition encompasses the fundamental aspects of existence, including emotional vulnerability, mortality, and the search for meaning and purpose in life. For many participants such as Carrie, Wally and Kate, ACC disrupted these dimensions profoundly, affecting not only themselves but also those close to them. Feelings of guilt, shame, self-blame, frustration, anger, sadness, grief, and loss were commonly reported.

In their efforts to endure and survive, some participants such as Kate and Emma focused on remaining strong and resilient both for themselves and those around them, at times underestimating or overlooking the emotional effects of their illness on loved ones. This disconnection often became apparent over time, as families and partners struggled alongside them. Grief emerged as a natural response to the losses associated with illness, including loss of health, independence, and anticipated futures (28). Participants described oscillating between complex emotional states, which allowed periods of reflection and adjustment as they sought to make sense of their changing identities and relationships. These findings highlight the importance of providing emotional and psychological support not only to individuals living with ACC but also to their families, as such support is integral to wellbeing, adaptation, and ongoing survival.

### 7.3 Theme 3: rollercoaster life

Betty, Lisa, Lucy, Rob and Tom described their lives as being abruptly disrupted and placed on hold following their diagnosis, with ACC profoundly reshaping their sense of self and daily existence. The embodiment of illness became central to their lived experience, influencing how they perceived reality, meaning, and their own humanity. Their diagnosis compelled them to confront fundamental questions about life, existence, and the human condition.

The suffering associated with ACC led many participants to experience a profound sense of loss, including the loss of comfort, stability, and certainty that had previously anchored their lives (29). Yet, within this suffering, resilience emerged as a transformative force. Participants described how enduring the challenges of cancer required them to rise above their former selves, to adapt, and to become “more” than they had been before their illness (30). Living with ACC demanded ongoing strength and adaptation, with resilience enabling individuals to withstand the physical, emotional, and existential burdens of the disease. This process of continual adjustment underscored the dynamic and often unpredictable nature of life with cancer.

#### 7.3.1 Enduring the disease burden

The participants, including Kevin, Mary, Emma and Muriel, described being confronted with the harsh realities of adjuvant therapy and its associated toxicities. Mitotane was frequently characterised as both a “potion and a poison”, reflecting its dual role in controlling the disease while imposing a substantial treatment burden. At the time of the interviews, several long-term survivors living with active incurable disease (e.g., Rob, Sue, and Stan) had remained on mitotane therapy for up to 10 years. The participants in remission also highlighted the life-sustaining role of mitotane, continuing treatment to reduce the risk of recurrence. Most participants reported that achieving and maintaining therapeutic mitotane levels significantly increased the burden of living with ACC. Those receiving mitotane often described a poorer quality of life than individuals who were no longer taking the medication, with notable impacts on their physical, emotional, and social functioning (14).

The change from symptoms of cortisol excess to the potentially life-threatening consequences of AI was described as overwhelming by Carrie and Emma. As highlighted in the earlier subtheme of living in uncertainty, even participants with extensive medical knowledge found this challenging. For example, Tom (described previously in subtheme 1.1) reported that it took nearly ten years to effectively manage adrenal crises due to the complex interplay between ACC and AI.

All participants managed their AI with hydrocortisone and described significant challenges in self-management, particularly during intercurrent illness. In such situations, they frequently relied on emergency care services, however, many reported that healthcare professionals lacked adequate knowledge of AI management in the context of ACC. This lack of awareness often exacerbated feelings of vulnerability and risk. As a result, some participants such as Carrie and Muriel described taking greater personal control over their care, learning to normalise or tolerate the side effects of treatment to continue with daily life. This adaptation, while necessary for survival, further illustrates the substantial physical and emotional burden carried by individuals living with ACC.

#### 7.3.2 Wading through the trauma

Trauma is commonly understood as an experience that is profoundly stressful, frightening, or distressing, and difficult for individuals to process or control. The accounts shared by the participants in this study reflected many of the defining features of trauma (e.g., Stan and Tom). However, not all participants felt able or willing to discuss their experiences openly. For some, including Wally and Mary, trauma was perceived as a source of shame or as an emotional burden that could distress others by confronting them with the reality of mortality.

Previous research suggests that post-traumatic stress disorder (PTSD) in cancer often functions as a chronic and ongoing stressor rather than a single, time-limited traumatic event (31). Many individuals living with cancer experiences persistent sadness and anxiety and may interpret trauma-related symptoms as normal emotional responses to illness rather than indicators of psychological distress (32). Studies using the Diagnostic and Statistical Manuel of Mental Disorders (DSM) criteria have reported that between 21% to 54% of individuals with cancers perceive their diagnosis as a traumatic stressor (33–35). During early stages of illness, participants described struggling to envisage a future free from disease, largely due to uncertainty surrounding prognosis and treatment outcomes. Consequently, some (e.g., Mary) chose to avoid dwelling on their experiences or attempted to normalise the physical and emotional effects of treatment as a coping strategy. This response appeared to serve as a protective mechanism, enabling them to continue functioning in the face of ongoing uncertainty and distress.

### 7.4 Theme 4: anchoring ways for survival

Some participants (e.g., Stan, Betty, Lucy, Jim and Wally) described needing periods of reflection to better understand themselves, their values, and the realities of coexisting with their disease. These periods allowed them to come to terms with their diagnosis, cultivate a more positive outlook, and prioritise aspects of life that they believed would improve their chances of survival. Through this process, many participants sought meaning and stability in the face of ongoing uncertainty. Stoicism emerged as a prominent coping strategy used to manage the psychological burden of ACC and to counter the perceived threat of the disease. Rooted in Hellenistic philosophy, Stoicism emphasises self-mastery, resilience, moral integrity, and the acceptance of circumstances beyond one’s control (36). Participants described how adopting stoic principles helped them to focus on what they could influence, enabling them to develop greater emotional regulation, mental strength, and spiritual resilience.

This philosophical orientation supported participants in finding meaning amid adversity and contributed to their ability to endure the physical and emotional challenges of living with ACC. Through these anchoring strategies, individuals were able to maintain a sense of purpose and agency despite the uncertainties associated with their illness.

#### 7.4.1 In the illusion of control

Participants such as Tom, Carrie and Betty described ongoing efforts to regain a sense of control while navigating the persistent challenges of living with ACC. Some framed this experience in terms of *liminality*, a transitional psychological and social state that exists between distinct phases of illness and recovery (37, 38). In the context of cancer, liminality reflects a sense of being “in-between”, a state that is often difficult for those without lived experience of illness to fully comprehend. This perceived ‘rite of passage’ through cancer brought about profound personal change, compelling participants to confront uncertainty, adapt to disruption, and re-evaluate their lives. In striving to regain control, participants sought to make sense of their circumstances and to reclaim agency over aspects of their lives that felt destabilised by illness. This reconstructed sense of control contributed to the development of resilience and offered participants a renewed sense of purpose and personal significance. It also enabled them to identify unmet needs, seek appropriate support, and address gaps in care, thereby helping them to navigate the complexities of living with ACC.

#### 7.4.2 Knowing me, knowing ACC

Knowing is a conscious and reflective process that enables individuals to understand their circumstances and make sense of how such knowledge may benefit them. For participants (e.g., Stan and James), awareness of ACC, and particularly the accuracy of survival statistics, was a significant and often challenging aspect of their experience. Over time, some participants, such as Sophie and Tom, came to accept their diagnosis and developed a deeper self-awareness that fostered resilience and informed future decision-making. Through reflection and lived experience, they gained a more nuanced understanding of their condition, allowing them to navigate uncertainty with greater confidence. This evolving understanding helped participants to make sense of the complexities of their illness, adapt to its challenges, and sustain hope throughout their cancer journey.

### 7.5 Theme 5: living and moving forward

The process of carrying on and moving forward with life continued as participants (e.g., Emma, Lucy, Lisa and James) gradually accepted and adjusted to living with ACC. Adaptation following a cancer diagnosis involved acknowledging and reconstructing self-esteem, understood as a sense of personal value and significance within one’s environment (39, 40). This recognition enabled individuals to view themselves as enduring and meaningful beings, rather than being defined solely by their illness (41). Through adapting to life with ACC, participants were able to resume previous routines or, in some cases, establish new directions and priorities. This process of adjustment reflected an active engagement with life despite ongoing uncertainty. Similar patterns have been reported in studies exploring the lived experiences of men with prostate cancer (42), young adults with cancer (43), and Thai women with breast cancer (44). The participants described learning to live and move forward by redefining meaning in their lives and resisting the tendency for ACC to become their defining identity. In doing so, they were able to maintain a sense of continuity, purpose, and agency beyond their illness.

#### 7.5.1 Finding perspectives and priorities

The participants (e.g., Tom, Sue and Jim) reflected deeply on their survival, with some attributing it to luck or even a miracle. A strong desire to live independently and maintain autonomy emerged across accounts. Hope was identified as essential and of paramount importance (e.g., Wally’s quote); however, many participants noted that it was largely absent from their early interactions with healthcare professionals. This finding aligns with previous research indicating that hope plays a central role in helping young people with cancer cope with the emotional burden of illness and treatment (43). For individuals with cancer, hope is often fostered through meaningful interactions with healthcare professionals and through relational, supportive care (45).

Through their experiences, participants described becoming kinder to themselves and developing greater self-compassion, which enabled them to find new direction and meaning in their lives. Self-compassion, defined as the ability to treat oneself with kindness during times of adversity (46, 47), was perceived as an important coping mechanism. Consistent with previous research, self-compassion, social support, and a sense of belonging were closely linked to resilience, particularly among individuals living with cancer (46). This renewed sense of purpose often brought participants closer to their loved ones, encouraging them to value relationships and everyday experiences more deeply. Many described a heightened appreciation of life and a shift in priorities, reflecting a process of self-actualisation (48). For Stan or those living with active disease, this perspective also provided time and psychological space to prepare for future challenges, enabling them to approach uncertainty with greater acceptance and resolve.

#### 7.5.2 Create moment of happiness

The participants were under no illusion that their cancers had been permanently cured. Rather than focusing on uncertainties beyond their control, they described a conscious decision to embrace the present and make the most of the opportunities available to them (e.g., Lisa, Kevin and Jim). Creating moments of happiness and meaning became a deliberate way of living, enabling them to experience a more fulfilling life alongside those closest to them. This shift in perspective allowed participants to prioritise connection, enjoyment, and purpose, despite the ongoing presence of illness. By focusing on what could be valued in the present, they were able to cultivate a sense of wellbeing and maintain hope in the face of uncertainty.

## 8 Discussion

Living with ACC was experienced as profoundly traumatic by participants and their loved ones. Their lives were described as resembling a “rollercoaster”, shaped by the life-threatening nature of the disease, uncertainty surrounding treatment options, substantial disease burden, inadequate care and support, loneliness, and disrupted relationships. Confronting ACC often forced participants to face mortality directly, an experience likened to looking at the sun, overwhelming and blinding in its intensity (48). In response, participants described having to fight for survival, regain control, and reconstruct their lives by redefining priorities and meaning.

The affective impact of ACC had significant consequences for emotional processing and psychological wellbeing. These emotional challenges compounded the physical burden of the disease and increased vulnerability to psychological distress, including suicidal ideation, particularly during the early stages following diagnosis. Previous research indicates that approximately 25% of individuals with Cushing’s syndrome experience suicidal ideation, with 10% attempting suicide (49). Individuals diagnosed with rare cancers, such as mesothelioma, pancreatic, and oesophageal cancers, have among the highest suicide risks, with rates 4.51-, 3.89-, and 2.65-fold higher, respectively, compared with more common cancers (50). The risk is greatest within the first six months following diagnosis. In this study, the emotional burden associated with Cushing’s syndrome, the toxicities of ACC treatment, and pervasive feelings of loneliness, helplessness, and hopelessness appeared to exacerbate this vulnerability.

Participants expressed a clear need for emotional support to help them cope with the burden of their illness, particularly younger individuals living with ACC. Many individuals felt that those around them struggled to fully understand the depth of their experience, which resulted in those with ACC offering emotional support to those around them. This finding underscores the significance of including close family members in comprehensive supportive care. Despite this, the current healthcare system was perceived as insufficient in addressing patients’ emotional and psychological needs, pointing to an urgent need for more comprehensive, person-centred support across healthcare settings.

Participants employed a range of coping strategies and consistently emphasised that, while they did not seek pity, they deeply valued compassion from healthcare professionals. Compassionate care has been defined as “a sensitivity to suffering in self and others with a commitment to try to alleviate and prevent it” (51). Although healthcare professionals may not always possess specialist expertise in ACC, compassionate practice can be strengthened through attentive listening, validation of patients’ experiences, and timely referral to specialist services when required (2, 5). Evidence suggests that clinicians who receive training in compassion, resilience, and psychological care deliver higher-quality care and achieve better patient satisfaction (47, 52).

Participants also highlighted the clinical challenges associated with mitotane therapy. Maintaining therapeutic serum levels above 14 mg/L was frequently associated with significant toxicity and reduced quality of life. Recent evidence has questioned the rigid application of this threshold, suggesting that lower, individualised dosing may achieve clinical benefit while reducing harm (53). This reinforces the importance of personalised treatment strategies and careful monitoring.

Support groups were viewed as valuable sources of emotional connection, though experiences were mixed. For some, hearing others’ stories was comforting; for others, it was emotionally overwhelming. Participants emphasised the need for readiness before engaging with peer support. Notably, learning of long-term survivors, such as an individual who had lived more than 21 years following an initial diagnosis of metastatic ACC, offered powerful hope and motivation. Hope emerged as a central driver of endurance and coping, underscoring its importance as a therapeutic construct in ACC survivorship care.

Finally, individuals requiring long-term mitotane therapy faced significant challenges in managing comorbidities, particularly adrenal insufficiency and pain. Difficulties with hydrocortisone metabolism and emergency management were common, and participants frequently felt ill-equipped to manage these complexities alone. These findings highlight the need for improved education, structured self-management support, and coordinated multidisciplinary care, particularly as increasing numbers of people are living longer with ACC.

## 9 Limitations of this study

This study involved a relatively small and culturally diverse sample of English-speaking participants from different countries. As such, the findings may not be readily generalisable, given the variation in healthcare systems, cultural contexts, and individual interpretations of lived experience. As with all qualitative research, the findings are also influenced by the subjective nature of participants’ accounts and the interpretive role of the researcher. Additionally, none of the participants were at the end-of-life stage of ACC at the time of the study. Consequently, the findings reflect the experiences of those living with and surviving ACC rather than those approaching end of life. Including non-English-speaking participants and individuals at later stages of the disease could provide a more comprehensive understanding of the full trajectory of living with ACC and enrich future research in this area.

## 10 Recommendations for clinical practice and research

To the authors’ knowledge, this study is the first to explore in depth the lived experiences, impacts, and meanings associated with living with ACC. The findings highlight the need for clinical care that extends beyond a focus on survival outcomes to include holistic support aimed at enhancing wellbeing. This includes supporting individuals to develop coping strategies and resilience to manage the physical and psychological demands of long-term, life-prolonging treatments. Accordingly, ACC care teams would benefit from training in psychological support to better address the complex emotional and psychosocial needs of this population.

Further research should adopt a comprehensive approach to understanding ACC and its associated conditions, including pre-diagnostic Cushing’s syndrome, treatment-induced adrenal insufficiency, and sexual dysfunction. Greater insight into how these factors influence diagnostic pathways, treatment experiences, quality of life, and survival outcomes is needed. Studies involving more diverse patient populations are essential to clarify determinants of wellbeing and survivorship across different demographic and healthcare contexts.

Further research is also required to evaluate psychosocial and counselling interventions tailored to people living with ACC, with the aim of improving emotional wellbeing and long-term outcomes. Interventions designed to foster adaptive coping should be informed by evidence from other cancer populations and chronic conditions and incorporated into integrated, ACC-specific care pathways. Given the central role of hope identified in this study, future work should explore its influence on psychological adjustment and survival in individuals with ACC. Targeted clinical strategies, including specialist nursing input, structured education on adrenal insufficiency, clinician training in mitotane management, and compassion-focused care, should be developed and rigorously evaluated. In particular, the management of adrenal insufficiency warrants further investigation, including alternative delivery methods for steroid replacement for patients who struggle with oral hydrocortisone. Finally, research exploring the experiences of families and caregivers is essential to better understand factors that promote resilience and strengthen support networks. Structured, person-centred care pathways should be initiated at diagnosis, with proactive referral to ACC-specific support organisations embedded within routine care, rather than relying on patients to seek support independently.

## 11 Conclusion

Individuals living with ACC face profound and multifaceted challenges that extend far beyond the physical consequences of the disease. Their experiences highlight the need for holistic, person-centred care that encompasses not only disease management but also to compassion, empathy, and sustained psychosocial support from healthcare professionals, family members, and the wider community.

Despite advances in treatment, the absence of precision medicine approaches for ACC often contributes to uncertainty and feelings of hopelessness among patients. Current healthcare systems are frequently ill-equipped to meet the specific needs of individuals with ACC, highlighting the need for further development of tailored services and more integrated care pathways. This should include greater involvement of General Practitioners in ongoing care to enhance continuity and support, despite the limited local expertise in managing ACC. Future research should focus on developing and evaluating interventions that address the psychosocial as well as the clinical needs of this underserved patient population.

## Data Availability

The raw data supporting the conclusions of this article will be made available by the authors, without undue reservation.
