# Supplementary material for: Hope is everything – an hermeneutic phenomenological study on the lived experiences of individuals with adrenocortical carcinoma
Source: Front Endocrinol (Lausanne). 2026 Jul 8;17:1829424. doi: 10.3389/fendo.2026.1829424 (PMC13388210; doi:10.3389/fendo.2026.1829424)
Supplement: Supplementary file 1 [file DataSheet1.pdf]

Supplementary 1: An interview topic guide for this study

**Opening question**	**Can you tell me when you were diagnosed with ACC and what symptoms you had at the time**
Listen for and follow-up on any of these aspects	• When were you diagnosed with ACC? What symptoms did you have at the time?<br>• How did you feel about being diagnosed with ACC?<br>• Can you tell me about your experience of living with ACC?<br>• Can you tell me about your daily life since you were diagnosed and if this has changed?<br>• What surgery and medical treatments did you have?<br>• How did the surgery and medications affect you in your daily life?<br>• What kind of treatments are you receiving now?<br>• What are the side-effects relating to your medication and treatments you are having?<br>• How are you affected by them?<br>• In what ways did they affect you?<br>• What did that mean for you?<br>• How did you make sense of what was happening?<br>• Did you have any side-effect? How did you manage your side-effects?<br>• Do you have other conditions related to your ACC?<br>• How do you manage these conditions?<br>• Which of these conditions affect you the most? (Look for AI, diabetes, skin issues etc)<br>• Did you try any methods to manage these conditions? What did you try? How effective were they?
**Probing questions**	**Did you talk to healthcare professionals (endocrinologist, endocrine nurse, oncologist, oncology nurse; about your ACC and its symptoms?**
Listen for and follow-up on any of these aspects	• To whom did you talk?<br>• What kind of information did you receive?<br>• Did you receive the advice you need? How useful was the advice?

	• Who else did you talk to about your ACC and your symptoms? Other members of healthcare professionals, members of your family, other patients?<br>• How did you find he information in managing your ACC and your symptoms?
**Probing questions**	**What other sources do you get your information from about managing your ACC and its symptoms & related conditions? (ACC Support UK, Adrenal Cancer Warriors, internet, Addisons Self Help, diabetes UK etc.)**
Listen for and follow-up on any of these aspects	• Do you have any contacts with friends/family diagnosed with ACC?<br>• Do you engage with a wider ACC community through social media or support groups for information?<br>• What other sources have you accessed to get your information about ACC and its related symptoms and conditions (such as AI, skin issues, mitotane, adjuvant therapy)?
**Probing questions**	**What information and support would help you with managing your ACC?**
Listen for and follow-up on any of these aspects	• In what form (such as verbal advice, factsheet, internet) should information and support should be provided? What is helpful to you?
**Probing questions**	**What are the positive and negative impacts of the treatment on you and your daily life?**
Listen for and follow-up on any of these aspects	• What are the obstacles and challenges you experienced in living with ACC?<br>• How do you manage and overcome these challenges?<br>• What are the circumstances which makes/would makes living with your ACC easier?
Closing question	• Would you like to add anything else?

Supplementary 2: Demographic and clinical characteristics of study participants

Name	Age	Sex	Relationship status	ACC status	Years with ACC	Tumour staging	Ki67% index	Surgery	Mitotane	Other adjuvant therapy	Country of residence at the time of interview
**Mary**	35	F	Married	Remission	4	II	6%	Yes	First 2 years	No	United Kingdom
**Sophie**	54	F	Married	Remission	4	II	14%	Yes	Last 4 years	No	USA
**Emma**	38	F	Single	Remission	4.5	III	25%	Yes	Last 4.5 years	No	Europe
**James**	69	M	Married	Remission	1.75	Not known	80%	Yes	First 8 months	Immunotherapy	United Kingdom
**Carrie**	52	F	Married	Remission	9.25	II	Not known	Yes	First 4 years	No	United Kingdom
**Jane**	31	F	Married	Active	0.75	IV	30%	Yes	Last 4 months	6 months EDP	United Kingdom
**David**	62	M	Married	Active	4	Not known	15%	Yes	None	No	Canada
**Tom**	39	M	Partnered	Remission	14	IV	Not known	Yes	First 10 years	No	United Kingdom
**Jack**	28	M	Single	Remission	6	III	50%	Yes	First 18 months	Radiotherapy	United Kingdom
**Rob**	43	M	Married	Active	9.5	Not known	Not known	Yes	Last 9.5 years	No	United Kingdom
**Kevin**	50	M	Married	Remission	5	IV	>30%	Yes	Last 5 years	No	Europe
**Lucy**	63	F	Married	Active	10	Not known	37%	Yes	Last 10 years	Radiotherapy	United Kingdom
**Jim**	67	M	Partnered	Remission	10	Not known	Not known	Yes	None	No	USA
**Sue**	61	F	Single	Remission	4.5	II	25%	Yes	None	No	Europe
**Kate**	41	F	Married	Remission	0.8	Not known	15%	Yes	Last 5 months	EDP	USA
**Lisa**	32	F	Single	Remission	5.5	II	12%	Yes	First 2 years	No	Europe
**June**	39	F	Married	Remission	1	IV	Not known	Yes	First 4 months	No	Nigeria
**Betty**	74	F	Widowed	Remission	21	Not known	Not known	No	First 18 years	No	United Kingdom
**Wally**	63	M	Married	Remission	13	IV	Not known	Yes	First year only	EDP + Ablation	United Kingdom
**Stan**	69	M	Married	Active	10	Not known	5%	Yes	Last 10 years	Ablation	United Kingdom
**Muriel**	50	F	Married	Remission	1.5	Not known	80%	Yes	Last 1.5 years	No	Australia

Tumour stage graded from I-IV **Ki67% is the tumour proliferation index** **Mitotane is the adrenolytic drug for ACC** **EDP stands for etoposide, doxorubicin, cisplatin**
